# Studying *Pseudomonas aeruginosa* pathogenesis: the need for more than just the PAO1 strain

**DOI:** 10.1128/spectrum.00665-25

**Published:** 2025-08-11

**Authors:** Laure David, Audrey Goman, Camille Pin, Frédéric Taieb, Priscilla Branchu, Eric Oswald

**Affiliations:** 1 IRSD, INSERM, INRAE, ENVT, Université de Toulouse 137668, Toulouse, France; 2 Service de Bactériologie-Hygiène, Centre Hospitalier Universitaire de Toulouse, Hôpital Purpan 36760, Toulouse, France; University of Florida, Gainesville, Florida, USA

**Keywords:** *Pseudomonas aeruginosa*, outer membrane vesicle, CprA, autophagy, inflammasome

## LETTER

We read with great interest the study by Ge *et al*. (1) titled “Inhibiting NLRP3 enhances cellular autophagy induced by outer membrane vesicles from *Pseudomonas aeruginosa*” (Microbiol Spectr. 2025 Mar 4;13(3):e0181924. doi: 10.1128/spectrum.01819-24). The authors propose that extracellular membrane vesicles (PA-OMVs) from *Pseudomonas aeruginosa* enhance autophagic flux in host cells, particularly through the modulation of the NLRP3 inflammasome pathway. However, we believe it is important to discuss these findings in the context of our own team’s recent work, which reveals a different perspective on the role of OMVs in autophagy and inflammasome activation.

In our studies, we demonstrated that the expression of the cytoplasmic short-chain dehydrogenase/reductase enzyme HlyF in *Escherichia coli*, or its ortholog CprA in *P. aeruginosa*, leads to the production of OMVs with a specific capacity to inhibit autophagic flux in epithelial cells and macrophages. This blockage of autophagy, in turn, exacerbates the activation of the noncanonical NLRP3 inflammasome pathway in host cells (2, 3). These findings contrast with those of Ge *et al.* (1), who report an increase in autophagic flux upon treatment with PA-OMVs.

This apparent discrepancy may be explained by the strain-specific differences in the production of OMVs. Ge *et al.* used the widely studied *P. aeruginosa* strain PAO1 (ATCC 15692), which, as we showed, produces a truncated form of CprA that is unable to generate OMVs with the same properties as those produced by other *P. aeruginosa* strains expressing full-length CprA (2). PAO1 is unique in its inability to produce OMVs that block autophagic flux, making it an outlier among *P. aeruginosa* strains. Therefore, we believe that studies relying solely on PAO1 may yield results that are not representative of the broader *P. aeruginosa* species, and caution should be exercised when generalizing findings to all PA-OMVs.

In light of these considerations, we suggest that the results reported by Ge *et al*. may be specific to PAO1 and should not be generalized to all OMVs produced by *P. aeruginosa*. We urge future studies to incorporate a broader range of *P. aeruginosa* strains to fully understand the complex interplay between autophagy, inflammasome activation, and the role of OMVs. Furthermore, given that CprA has the capacity to modify the lipids of the *P. aeruginosa* outer membranes (2), it is very likely that the role of CprA goes beyond the simple production of OMV blocking autophagic flux and that here again it is necessary to use strains of *P. aeruginosa* that produce a functional CprA protein.

We appreciate the authors’ valuable contribution to this field and look forward to further discussions on the molecular mechanisms underlying bacterial-host interactions.
